# Thermal Bridges on Building Rooftops

**DOI:** 10.1038/s41597-023-02140-z

**Published:** 2023-05-10

**Authors:** Zoe Mayer, James Kahn, Markus Götz, Yu Hou, Tobias Beiersdörfer, Nicolas Blumenröhr, Rebekka Volk, Achim Streit, Frank Schultmann

**Affiliations:** 1 Karlsruhe Institute of Technology, Institute for Industrial Production, 76187 Karlsruhe, Germany; 2 Helmholtz AI, Karlsruhe, Germany; 3 Karlsruhe Institute of Technology, Steinbuch Centre for Computing, 76344 Eggenstein-Leopoldshafen, Germany; 4 Western New England University, Department of Construction Management, Springfield, MA 01119 USA; 5 Carnegie Mellon University, Civil and Environmental Engineering Department, Pittsburgh, PA 15213 USA; 6 Helmholtz Metadata Collaboration, Karlsruhe, Germany

**Keywords:** Civil engineering, Computer science

## Abstract

Thermal Bridges on Building Rooftops (TBBR) is a multi-channel remote sensing dataset. It was recorded during six separate UAV fly-overs of the city center of Karlsruhe, Germany, and comprises a total of 926 high-resolution images with 6927 manually-provided thermal bridge annotations. Each image provides five channels: three color, one thermographic, and one computationally derived height map channel. The data is pre-split into training and test data subsets suitable for object detection and instance segmentation tasks. All data is organized and structured to comply with FAIR principles, i.e. being findable, accessible, interoperable, and reusable. It is publicly available and can be downloaded from the Zenodo data repository. This work provides a comprehensive data descriptor for the TBBR dataset to facilitate broad community uptake.

## Background & Summary

About 30% of global final energy consumption and 27% of total energy sector emissions stem from building operations. After a short drop during the COVID-19 pandemic, emissions and energy consumption are both now above their pre-COVID level of 2019, showing that no late reduction trend has started^1^.

A major field for reducing energy consumption for building operations is the improvement of building envelopes, which is critical for reductions in heating and cooling intensity^2^. A thermal bridge is a discontinuity of a building’s envelope, whose thermal properties differ fundamentally from the thermal properties of the adjacent enveloping surface^3^. With increasing demands on the quality of building envelopes, the minimization of thermal bridges is becoming ever more important, since losses from thermal bridges can account for up to one third of a building’s transmission heat loss^4,5^. Beyond increased energy consumption, thermal bridges can lead to a wide range of problems, from the risk of condensation and mold infestation^6^, to a reduced comfort that occurs due to cold inner surfaces of a building^7^. In summer, thermal bridges lead to increased heat absorption by buildings and thus can increase the need for air conditioning^3^.

For the detection of thermal bridges of building envelopes, thermography can be reliably used^8^. In recent years, not only individual buildings, but also buildings in their urban context have gained importance for developing adequate retrofit strategies. The New Urban Agenda of the United Nations (UN) puts a spotlight on policies affecting urban structures at all appropriate levels recognizing that building design is one of the “greatest drivers of cost and resource efficiencies”^9^. When studying building stocks in cities, city districts, and villages, thermographic images can be collected with Unmanned Aerial Vehicles (UAVs/drones)^10,11^. Thermography with drones is especially advantageous because it saves time, resources, and is scalable for large areas compared to classical thermography with static cameras^10^. UAV-based thermographic systems are particularly beneficial when examining rooftops, since recordings with hand-held cameras are difficult. Previously, rooftop inspections with thermography had to be carried out on the basis of on-site inspections at night which are particularly labor-intensive, dangerous, and unable to achieve the same coverage feasible with drones^12^.

To evaluate large number of thermographic images collected in urban areas, the manual processing of images is time-consuming. The detection of thermal bridges can be automated, but is not trivial. Currently, approaches for automated thermal bridge detection work mostly with temperature threshold values and pattern recognition^13–16^. It is, however, difficult to find threshold values that can be generally applied to all types of thermal bridges^17^. Patterns and temperatures differ depending on the materials and building components where thermal bridges occur, on environmental conditions, and on recording settings. For example for windows, temperatures on thermographic images appear cooler due to high levels of reflection of glass surfaces^18^. Furthermore, misinterpretations, e.g. caused by open windows, can occur with simple threshold methods. Deep learning methods, which can overcome the aforementioned problems, may provide better results, but require annotated image datasets.

In this data descriptor, we present the Thermal Bridges on Building Rooftops (TBBR) dataset. To the best of our knowledge it is the first comprehensive aerial thermographic image dataset, which also provides height mapping information while also being fully annotated for district-scale segmentation of thermal bridges on building rooftops. It is organized and structured according to the FAIR principles^19^, i.e. being findable, accessible, interoperable and reusable.

The remainder of the data descriptor is organized as follows: the Methods section describes the environmental conditions and methodological approach in recording the TBBR dataset. Data Records details the organization of the data, including file formats, how the data has been preprocessed and curated, as well as how to obtain it from a publicly available data repository. In the Technical Validation section we highlight data quality aspects of TBBR. Finally, the Usage Notes sections sketches current and prospective use case scenarios for the data with an emphasis on (semi-)automated thermal bridge object detection and instance segmentation.

## Methods

**Fig. 1:**
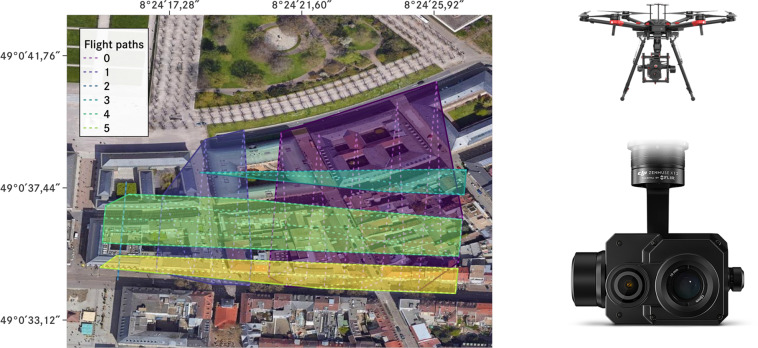
Geo-located map of drone flyover regions (left, WGS 84 coordinate system, source: Google Maps), DJI M600 drone (upper right), and Zenmuse XT2 camera with a FLIR Tau 2 thermal sensor (lower right). Dashed lines show the flight paths of the drone, polygons the photographed regions. Numbers correspond to identifier of each flight paths, e.g. 2 for Flug1_10**2** (see Data Records section below). Image source for the drone and camera: © DJI.

The raw images for our dataset were recorded with a Zenmuse XT2 visual (RGB) and a FLIR Tau 2 (thermal, https://flir.netx.net/file/asset/15598/original/) camera (see Table 1 for details) on a DJI M600 drone (https://www.dji.com/de/matrice600). They were recorded at flight heights between 60–80 m above ground with a flight speed of $1\frac{{\rm{m}}}{{\rm{s}}}$ and contain GPS information. The images cover six large blocks of around 20 buildings per block recorded in the city center of the German city Karlsruhe with a total fly-over area of roughly 48500 m^2^ (see Fig. 1). Because of a high overlap rate of the images, the same buildings are on average recorded from different angles in different images about 20 times. All images were recorded during drone flights on Tuesday 19th March 2019 from 7am to 8am (UTC + 02:00). At this time, temperatures were between 3.78 °C and 4.97 °C, and humidity between 80% and 98%. There was no rain on the day of the flights, but there was $2.3\frac{{\rm{mm}}}{{{\rm{m}}}^{2}}$ 48 hours beforehand. For all images, an exposure time of 1/100 s and ISO speed rating of 128 was used. For recording the thermographic images, an emissivity of 1.0 and an aperture of F1 was set. For the RGB images, an aperture of F1.8 was used. The global radiation during this period was between $38.59\frac{{\rm{W}}}{{{\rm{m}}}^{{\rm{2}}}}$ and $120.86\frac{{\rm{W}}}{{{\rm{m}}}^{{\rm{2}}}}$. No direct sunlight can be seen visually on any of the recordings. Further environmental conditions are shown in Table 2. We do not provide information on the recorded buildings’ internal temperatures, for estimates we refer readers to the corresponding German DIN standards^20^.

**Table 1:** Technical specifications of the cameras used in recording the TBBR raw data.

Camera	Spectrum (μm)	Image Resolution (px)	FOV (°)	Focal Length (mm)	Format
Zenmuse XT2 (RGB)	0.4–0.7	4000 × 3000	57.12 × 42.44	8	TIFF
FLIR Tau 2 (thermal)	7.5–13.5	640 × 512	45.00 × 37.00	13	TIFF

As the thermal camera is less than one year since purchase, it is still factory calibrated (see https://www.flir.co.uk/support-center/surveillance/infrared-camera-calibration/).

**Table 2:** Environmental conditions during the fly over on 2019-03-19 as measured by the closest weather station in Rheinstetten N 48°58′21.4″N 8°19′48.4″E (WGS 84 coordinate system, source: DWD OpenData at https://opendata.dwd.de/climate_environment/CDC/observations_germany/climate/hourly/).

Time	Cloud Cover	Pressure (hPa)	Visibility (m)	Wind Speed ($\frac{{\rm{m}}}{{\rm{s}}}$)	Wind Direction (°)
7am UTC+02:00	overcast	1012.0	29680	0.7	280
8am UTC+02:00	overcast	1012.7	34430	1.1	90

The full set of raw images captured contained a total of 5698 images before preselection^21^. Preselection involved the removal of all blurry images, e.g. due to rapid movement or turning of the drone, and all images containing no visible thermal bridges. After preselection a total of 926 images remained.

The RGB and thermal drone images were fused with a computed height map. All images were converted to a uniform format of 4000 × 3000 px, aligned, and cropped to 3370 × 2680 px to remove empty borders. The annotations only include thermal bridges that are visually identifiable with the human eye. Because of the aforementioned image overlap, each thermal bridge is annotated multiple times from different angles. For the annotation of the thermal images the image processing program VGG Image Annotator from the Visual Geometry Group, version 2.0.10^22^, was used. The thermal bridge annotations are outlined with polygon shapes. These polygon lines were placed as close as possible but outside the area of significant temperature increase. If a detected thermal bridge was partially covered by another building component located in the foreground, the thermal bridge was also marked across the covering in case of minor coverings. Adjacent thermal bridges, which affect different rooftop components, were annotated separately. For example, a window with poor insulation of the window reveal located in the area of a poorly insulated roof is annotated individually. There is no overlap between annotated areas. While each image contains annotations, they also include thermal bridges present that are not annotated due to not being clearly identifiable, e.g. too small for accurate identification or unclear due to the camera perspective.

### Image preparation

**Fig. 2:**
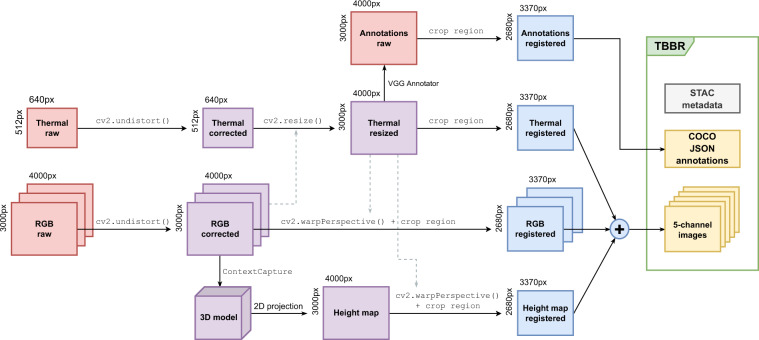
Image registration and alignment procedure.

The image registration and alignment procedure is shown in Fig. 2. The procedure involves three main steps:

1. distortion correction,
2. registration and alignment,
3. cropping and stacking.

The distortion correction procedure used was that established in previous works^23,24^. In short, a reference image was used to determine distortion coefficients, cv2.getOptimalNewCameraMatrix() to find a new camera matrix, and cv2.undistort() to correct distortion. All mentioned processing functions are part of the computer vision programming library OpenCV^25^.

Image registration and alignment was then performed by transforming the RGB and height map images onto the thermal images, as the annotation of thermal bridges was performed on these. A homography matrix was calculated using a total of 316 coordinate pairs from 21 RGB and thermal images. This homography matrix was then used to transform all RGB images in the dataset. Since the height map was created from the RGB images, we also used this homography matrix to transform the height map images.

The final cropping and stacking was performed to create the 5-channel images of the TBBR dataset, output in the NumPy format^26^. Images are cropped to 3370 × 2680 px to remove large black borders present in thermal images, and subsequently stacked into the channel order [B, G, R, Thermal, Height].

### Computation of the height map

Due to the high overlap of images, we can extract similarities from feature points identified in each image and conduct photogrammetry. Photogrammetry allows estimation of the three-dimensional coordinates of points on an object in a generated 3D space involving measurements made on images taken with a high overlap rate. Therefore, we can use this technique to create a 3D point cloud model of the recorded region.

We used the ContextCapture software to perform photogrammetry on the TBBR dataset. ContextCapture provides users with intermediate information necessary to obtain each image’s estimated 3D coordinates and orientation^23,24^. This information allowed estimation of the distance between points in 3D and 2D spaces and to project points from the 3D to the 2D space to generate the height maps. The resulting 2D height map image pixels show the z-axis value (vertical height) of the corresponding 3D point cloud model points, normalized to the 8-bit range of the lowest 3D model point (0) and the drone (255).

## Data Records

**Fig. 3:**
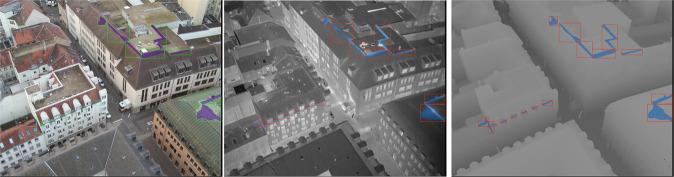
Example image from the TBBR dataset (Flug_100, ID 523) showing the different channels, RGB (left), thermal (center), and height map (right), including overlaid annotations.

The Thermal Bridges on Building Rooftops (TBBR) data is publicly available on Zenodo^27^ and is licensed under Creative Commons Attribution 4.0 International (http://creativecommons.org/licenses/by/4.0/). The 926 images in the dataset are made available as a series of compressed archive files totaling 68.5GB. Each compressed archive file corresponds to one of the six flight paths, named Flug1_100 to Flug1_105 respectively (the word “Flug” means flight in German). The archives contain NumPy^26^ files (one per image) of shape (2680,3370,5), where the final dimension is the color channel in the format [B, G, R, Thermal, Height]. An example image (Flug_100, ID: 523) is depicted in Fig. 3. Archives were compressed using ZStandard compression^28^. They can be decompressed by utility software programs, e.g. tar or unzstd. Corresponding annotations are provided in the COCO JSON format^29^, which were automatically generated by the VGG Image Annotator.

One of TBBR’s main design objectives was to facilitate (semi-)automated thermal bridges pattern detection algorithms^30^ (see Usage Notes). In accordance, the data is pre-split into train and test subsets with 723 (5614) and 203 (1313) images (annotations), respectively. There is one annotation COCO JSON for each subset, i.e. one for training (Flug1_100Media to Flug1_104Media) and one for test (Flug1_105Media) data. The latter block is used as a hold-out test dataset to standardize out-of-sample generalization performance assessment.

The experimental metadata was structured with the Spatio Temporal Asset Catalog (STAC) (https://stacspec.org/en) specification family. This specification is used to provide a standardized way for describing geo-spatial assets. It defines related JSON object types of Item, Catalog, and Catalog, extending Collection as the basis. Moreover, STAC objects can be extended with other specifications and enable a mechanism to provide additional metadata. Such an approach addresses the relevance for a common understanding of experimental metadata, which is ideally a widely accepted standard^31^.

The STAC Collection JSON object Flug1_collection_stac_spec provides information about the recorded images and the environmental conditions during recordings. It also contains information about the overall bounding box of the entire area in which images were recorded. It links to related STAC Item JSON objects containing information about the recorded city blocks and the cameras. The objects for the six flight paths, i.e. Flug1_100_stac_spec, Flug1_101_stac_spec, Flug1_102_stac_spec, Flug1_103_stac_spec, Flug1_104_stac_spec, Flug1_105_stac_spec, contain the GeoJSON^32^ geometry of the respective block and the corresponding bounding box.

The objects containing the camera information, named Flug1_camera1_stac-spec for the RGB camera and Flug1_camera2_stac-spec for the Thermal camera, are based on an existing STAC extension for camera related metadata. All STAC Item objects have a link to the Flug1_collection_stac_spec Collection object.

Metadata of the archived NumPy files for each image was structured using the Data Package schema from the Frictionless Standards (https://specs.frictionlessdata.io). This standard describes a collection of data files. Therefore, metadata about all containerized NumPy files of the six flight paths is provided within a JSON-based file, named Flug1_100-105_frictionless_standards.

All files are represented in a standardized way as FAIR Digital Objects (FAIR DOs) to enable machine actionable decisions on the data in the spirit of the FAIR principles^33^. This representation further facilitates reproducibility of experiments performed using TBBR and the detection of data errors^34^. Thus, each file deposited in Zenodo (10.5281/zenodo.7022736)^27^ was assigned a Persistent Identifier (PID), which is resolvable with the Handle.Net Registry (HNR) (https://www.handle.net/). The full list of PIDs are listed in the TBBR Zenodo dataset description^27^.

## Technical Validation

The visual identification process and description of thermal bridges on building rooftops was based on typical patterns described in German DIN standards^35–37^ and thermal infrared inspections^38^. We note, however, that the interpretation of thermal images for building audits is currently always performed by human operators, which involves a high level of subjectivity^13^.

Thermal bridges occur on different parts of rooftops. Table 3 provides an overview about the different roof types and rooftop components where thermal bridges were annotated.

**Table 3:** TBBR annotation and component overview.

	Train	Test	Total
Annotated images	723	203	926
Total annotations	5614	1313	6927
Average annotations per image	7.8	6.5	7.5
**Rooftop shape**	**No. of annotations**		
Steep roof	3939	895	4834
Flat roof	524	379	903
Mixed shape	1151	39	1190
**Rooftop component**	**No. of annotations**		
Rooftop surfaces	437	185	622
Component connections (dormers, ridges, valleys, gables, eaves lines, etc.)	3977	842	4819
Cantilevers (cantilever walls, attics, cantilever floor slabs, etc.)	640	149	789
Windows (reveals, lintels, parapets, dome lights, etc.)	560	137	697

All preselected images were first manually annotated by a single industrial engineer. Then, following the two-person principle, all annotations were subsequently reviewed independently by an expert supervisor and corrected when necessary.

**Fig. 4:**
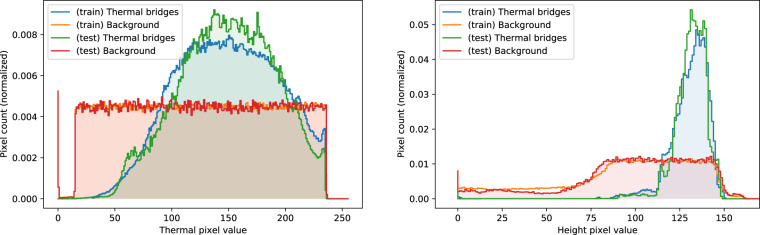
Histograms of thermal (left) and height map (right) pixel values of thermal bridges and background for both the train and test subsets within their 8-bit channel ranges of [0, 255]. Note that the height map values have been truncated slightly above their maximum at 170 for visual clarity. The zero valued pixel peaks arises from slight (~20 pixels) black borders remaining on the right side of images after cropping.

We qualitatively compare the distributions of thermal and height map values of thermal bridges and background between the train and test subsets. Figure 4 shows the histograms of both distributions within their 8-bit channel ranges of [0,255]. As expected, we observe a uniform distribution of thermal values across background pixels, while there is a distinct peak in warmer pixels for thermal bridges. Similarly, we see the presence of thermal bridges on rooftops only being reflected in the large height map values of thermal bridges, while background pixels are distributed uniformly both at the building level, and to a lesser extent at street level.

**Fig. 5:**
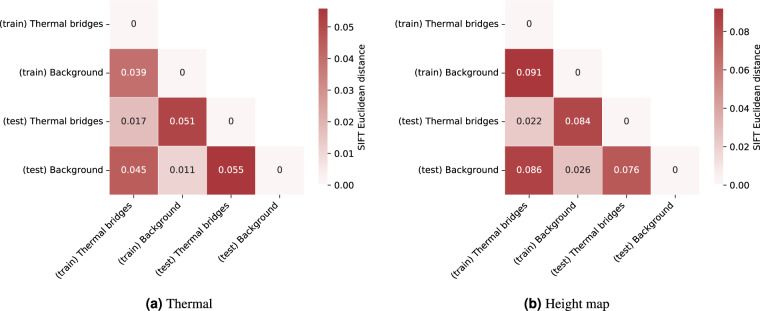
Euclidean distances between SIFT descriptors for thermal bridges and background annotations between train and test subsets.

To quantitatively compare annotated distributions, we use scale invariant feature transform (SIFT) descriptors^39^ which has been shown to have a good general robustness across a range of image transformations^40^, e.g. affine transformations, scale changes, and rotations, making it an appropriate comparison for thermal bridge images of rooftops from various distances and angles. Figure 5 shows the average Euclidean distances between all 128 SIFT descriptors for annotated thermal bridges and background pixels across the train and test subsets. We observe a small distance between like classes across both train and test subsets, and larger relative distances for unlike classes, indicating that annotated regions contain distinct features from background in a consistent manner.

## Usage Notes

**Fig. 6:**
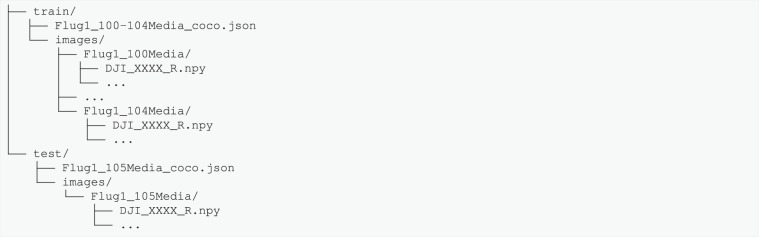
Recommended folder structure for TBBR dataset.

The annotation files contain relative paths to the NumPy files. We recommend the folder structure shown in Fig. 6 for usage of TBBR in conjunction with computer vision libraries such as Detectron2^41^ or MMDetection^42^, or with the provided TBBRDet library (see Code Availability).

For image analysis pipelines we recommend to standardize the images, i.e. center it to 0 mean with a standard deviation of 1, to make the different channel ranges of the image data comparable:

$${Z}_{(w\times h,c)}=\frac{{I}_{(w\times h,c)}-{\overline{I}}_{(c)}}{\sigma {(I)}_{(c)}},$$

where *Z* is the transformed data, *I* the input images, overlines are mean values and *σ* the standard deviation, subscripts denote shapes of the data. For ease-of-use, we have precomputed the resulting values:

$${\overline{I}}_{(5)}=[130.0,135.0,135.0,118.0,118.0]\quad \quad \sigma {(I)}_{(5)}=[44.0,40.0,40.0,30.0,21.0].$$

## Data Availability

Processing code is publicly available and can be found at https://github.com/Helmholtz-AI-Energy/TBBRDet. The software is licensed under the Revised Berkley Software Distribution (BSD-3) license (https://opensource.org/licenses/BSD-3-Clause). All scripts are implemented with the Python (v.3.6.8) programming language^43^ and utilize the PyTorch (v.1.10.2) machine learning framework^44^. Conceptually, the software provides the following functionalities: **VGG annotation to COCO JSON converter** implementing fully automatic conversion from the annotation format generated during the manual labeling process into the COCO JSON format archived on Zenodo. **Dataset mappers** for the Detectron2 and MMDetection libraries implementing random-access collections to individual images and corresponding annotations. These are necessary for enabling the loading of five-channel images in each library. Data may be augmented by arbitrary transformations during the loading procedure. **Model configuration** for all Detectron2 and MMDetection experiments performed in related works. **Training/evaluation scripts** for performing training and evaluation of neural networks for both Detectron2 and MMDetection. **Dataset/experiment utilities** for exploring the dataset, calculating image normalization coefficients, combining model scores, and calculating SLURM workload manager system^45^ statistics (consumed energy, runtime, etc.). For creating, updating, and validating the FAIR DOs, the Typed PID Maker was used. This is a component of the FAIR DO Lab for working on FAIR DO tasks, which is found at https://github.com/kit-data-manager/FAIR-DO-Lab.

## References

[1] International Energy Agency (IEA). Tracking Buildings 2022. Tech. Rep., International Energy Agency (IEA), Paris (2022).

[2] International Energy Agency (IEA). Building Envelopes. Tech. Rep., International Energy Agency (IEA), Paris (2021).

[3] Ge H, Baba F (2015). Dynamic Effect of Thermal Bridges on the Energy Performance of a Low-Rise Residential Building. Energy and Buildings.

[4] Schild, K. *Wärmebrücken* (Springer Fachmedien, Wiesbaden, 2018).

[5] Theodosiou TG, Papadopoulos AM (2008). The Impact of Thermal Bridges on the Energy Demand of Buildings with Double Brick Wall Constructions. Energy and Buildings.

[6] Fantucci S, Isaia F, Serra V, Dutto M (2017). Insulating coat to prevent mold growth in thermal bridges. Energy Procedia.

[7] Kalamees, T., Korpi, M., Eskola, L., Kurnitski, J. & Vinha, J. *The Distribution of the Air Leakage Places and Thermal Bridges in Finnish Detached Houses and Apartment Buildings* (Danish Society of Engineers, IDA, 2008).

[8] Kylili A, Fokaides PA, Christou P, Kalogirou SA (2014). Infrared thermography (IRT) applications for building diagnostics: A review. Applied Energy.

[9] General Assembly of the United Nations. New Urban Agenda: Resolution Adopted by the General Assembly (2016).

[10] Ma, L., Li, M., Tong, L., Wang, Y. & Cheng, L. Using Unmanned Aerial Vehicle for Remote Sensing Application. In *2013 21st International Conference on Geoinformatics*, 1–5, 10.1109/Geoinformatics.2013.6626078 (2013).

[11] Mayer, Z., Kahn, J., Hou, Y. & Volk, R. AI-based thermal bridge detection of building rooftops on district scale using aerial images. In *EG-ICE 2021 Workshop on Intelligent Computing in Engineering. Ed.: Jimmy Abualdenien, André Borrmann, Lucian-Constantin Ungureanu, Timo Hartmann*, 497 (2021).

[12] Zhang J, Jung J, Sohn G, Cohen M (2015). Thermal Infrared Inspection of Roof Insulation Using Unmanned Aerial Vehicles. ISPRS - International Archives of the Photogrammetry, Remote Sensing and Spatial Information Sciences.

[13] Garrido I, Lagüela S, Arias P, Balado J (2018). Thermal-Based Analysis for the Automatic Detection and Characterization of Thermal Bridges in Buildings. Energy and Buildings.

[14] Macher, H., Landes, T. & Grussenmeyer, P. Automation of Thermal Point Clouds Analysis for the Extraction of Windows and Thermal Bridges of Building Facades. In *The International Archives of the Photogrammetry, Remote Sensing and Spatial Information Sciences*, vol. XLIII-B2-2020, 287–292, 10.5194/isprs-archives-XLIII-B2-2020-287-2020 (Copernicus GmbH, 2020).

[15] Rakha T, Liberty A, Gorodetsky A, Kakillioglu B, Velipasalar S (2018). Heat Mapping Drones: An Autonomous Computer-Vision-Based Procedure for Building Envelope Inspection Using Unmanned Aerial Systems (UAS). Technology: Architecture + Design.

[16] Mirzabeigi, S. & Razkenari, M. Automated Vision-Based Building Inspection Using Drone Thermography. In *20th Annual New York State Green Building Conference*, 737–746, 10.1061/9780784483961.077 (American Society of Civil Engineers, 2022).

[17] Kim C, Choi J-S, Jang H, Kim E-J (2021). Automatic Detection of Linear Thermal Bridges from Infrared Thermal Images Using Neural Network. Applied Sciences.

[18] Flores Larsen S, Hongn M (2014). Determining the infrared reflectance of specular surfaces by using thermographic analysis. Renewable Energy.

[19] Wilkinson MD (2016). The FAIR Guiding Principles for scientific data management and stewardship. Scientific data.

[20] Deutsches Institut für Normung e.V. (DIN). DIN 4108-2:2013-02, Thermal protection and energy economy in buildings - Part 2: Minimum requirements to thermal insulation. Tech. Rep., Beuth Verlag GmbH. 10.31030/1929159 (2013).

[21] Kahn, J. *et al*. Hyperspectral (RGB + Thermal) drone images of Karlsruhe, Germany - Raw images for the Thermal Bridges on Building Rooftops (TBBR) dataset, 10.5281/zenodo.7360996 (2022).

[22] Dutta, A. & Zisserman, A. The VIA Annotation Software for Images, Audio and Video. In *Proceedings of the 27th ACM International Conference on Multimedia*, MM ‘19, 10.1145/3343031.3350535 (ACM, New York, NY, USA, 2019).

[23] Hou, Y., Volk, R., Chen, M. & Soibelman, L. Fusing tie points’ RGB and thermal information for mapping large areas based on aerial images: A study of fusion performance under different flight configurations and experimental conditions. *Automation in Construction* **124**, 10.1016/j.autcon.2021.103554 (2021).

[24] Hou, Y., Chen, M., Volk, R. & Soibelman, L. Investigation on performance of RGB point cloud and thermal information data fusion for 3D building thermal map modeling using aerial images under different experimental conditions. *Journal of Building Engineering* **45**, 10.1016/j.jobe.2021.103380 (2022).

[25] Bradski G (2000). The OpenCV library. Dr. Dobb’s Journal: Software Tools for the Professional Programmer.

[26] Harris CR (2020). Array programming with NumPy. Nature.

[27] Mayer Z (2022). Zenodo.

[28] Collet, Y. & Kucherawy, M. RFC 8878: Zstandard Compression and the ‘application/zstd’ Media Type. Tech. Rep., Internet Engineering Task Force (IETF). 10.17487/RFC8878 (2021).

[29] Lin, T.-Y. *et al*. Microsoft COCO: Common Objects in Context. In *European conference on computer vision*, 740–755, 10.1007/978-3-319-10602-1_48 (Springer, 2014).

[30] Mayer Z (2023). Deep learning approaches to building rooftop thermal bridge detection from aerial images. Automation in Construction.

[31] Musen MA (2022). Without appropriate metadata, data-sharing mandates are pointless. Nature.

[32] Butler, H. *et al*. The GeoJSON Format. Request for Comments RFC 7946, Internet Engineering Task Force. 10.17487/RFC7946 (2016).

[33] Schwardmann, U. Digital objects – fair digital objects: Which services are required? *Data Science Journal* **19**, 10.5334/dsj-2020-015 (2020).

[34] Berberi, I. & Roche, D. G. No evidence that mandatory open data policies increase error correction. *Nature Ecology & Evolution* 1–4, 10.1038/s41559-022-01879-9 (2022). 10.1038/s41559-022-01879-9 36109655

[35] Deutsches Institut für Normung e.V. (DIN). DIN EN 13187:1999-05, Thermal performance of buildings - Qualitative detection of thermal irregularities in building envelopes - Infrared method (ISO 6781:1983, modified); German version EN 13187:1998. Tech. Rep., Beuth Verlag GmbH. 10.31030/8035327 (1999).

[36] Deutsches Institut für Normung e.V. (DIN). DIN EN ISO 10211:2018-03, Thermal bridges in building construction - Heat flows and surface temperatures - Detailed calculations (ISO 10211:2017); German version EN ISO 10211:2017. Tech. Rep., Beuth Verlag GmbH, 10.31030/2522431 (2018).

[37] Deutsches Institut für Normung e.V. (DIN). DIN 4108 Beiblatt 2:2019-06, Thermal insulation and energy economy in buildings; Supplement 2: Thermal bridges - Examples for planning and performance, with CD-ROM. Tech. Rep., Beuth Verlag GmbH. 10.31030/3054799 (2019).

[38] Balaras CA, Argiriou AA (2002). Infrared thermography for building diagnostics. Energy and Buildings.

[39] Lowe DG (2004). Distinctive Image Features from Scale-Invariant Keypoints. International Journal of Computer Vision.

[40] Mikolajczyk K, Schmid C (2005). A performance evaluation of local descriptors. IEEE Transactions on Pattern Analysis and Machine Intelligence.

[41] Wu, Y., Kirillov, A., Massa, F., Lo, W.-Y. & Girshick, R. Detectron2 (2019).

[42] Chen, K. *et al*. MMDetection: Open MMLab detection toolbox and benchmark. *arXiv preprint arXiv:1906.07155*, (2019).

[43] Van Rossum, G. & Drake, F. L. *Python 3 Reference Manual* (CreateSpace, Scotts Valley, CA, 2009).

[44] Paszke, A. *et al*. Pytorch: An imperative style, high-performance deep learning library. In Wallach, H. *et al*. (eds.) *Advances in Neural Information Processing Systems* **32**, 8024–8035 (Curran Associates, Inc., 2019).

[45] Yoo, A. B., Jette, M. A. & Grondona, M. SLURM: Simple Linux Utility for Resource Management. In Feitelson, D., Rudolph, L. & Schwiegelshohn, U. (eds.) *Job Scheduling Strategies for Parallel Processing*, Lecture Notes in Computer Science, 44–60, 10.1007/10968987_3 (Springer, Berlin, Heidelberg, 2003).

